# Clinical and Immunological Impacts of Latent Toxoplasmosis on COVID-19 Patients

**DOI:** 10.7759/cureus.45989

**Published:** 2023-09-26

**Authors:** Samar Habib, Eman Hamza, Randa El-Gamal, Nessma A Nosser, Wafaa A Aboukamar, Sherehan Abdelsalam, Ali Sobh, Mohamed Elegezy, Mohamed Elbayoumy, Waleed Eldars, Khaled Elmasry, Marwa H Elnagdy

**Affiliations:** 1 Department of Medical Parasitology, Faculty of Medicine, Mansoura University, Mansoura, EGY; 2 Department of Medical Biochemistry and Molecular Biology, Faculty of Medicine, Mansoura University, Mansoura, EGY; 3 Department of Medical Biochemistry and Molecular Biology, Faculty of Medicine, Horus University, New Damietta, EGY; 4 Medical Experimental Research Center (MERC), Faculty of Medicine, Mansoura University, Mansoura, EGY; 5 Department of Clinical Pathology, Faculty of Medicine, Mansoura University, Mansoura, EGY; 6 Department of Community Medicine, Faculty of Medicine, Mansoura University, Mansoura, EGY; 7 Department of Pediatrics, Mansoura University Children's Hospital, Faculty of Medicine, Mansoura University, Mansoura, EGY; 8 Department of Tropical Medicine, Faculty of Medicine, Mansoura University, Mansoura, EGY; 9 Department of Gastroenterology and Hepatology, King Saud Medical City, Riyadh, SAU; 10 Department of Medical Microbiology and Immunology, Faculty of Medicine, Mansoura University, Mansoura, EGY; 11 Department of Basic Medical Sciences, Faculty of Medicine, New Mansoura University, New Mansoura, EGY; 12 Department of Oral Biology and Diagnostic Sciences, Dental College of Georgia, Augusta University, Augusta, USA; 13 Department of Human Anatomy and Embryology, Faculty of Medicine, Mansoura University, Mansoura, EGY

**Keywords:** parasitic infections, cytokines, covid-19, immune-modulators, toxoplasma gondii

## Abstract

Background

Parasites are well-known immune-modulators. They inhibit some aspects of the immune system to ensure persistence inside the host for a long time; meanwhile, they stimulate other immune aspects to assure the survival of the host. Wide variations in the severity of coronavirus disease 2019 (COVID-19) among developed and developing countries were reported during the COVID-19 pandemic. Parasitic infections, including *Toxoplasma gondii *(*T. gondii*), were claimed to contribute to such variations.

Methods

To explore a possible relationship between latent toxoplasmosis and COVID-19 severity, our study included 44 blood samples from moderate/severe COVID-19 patients, who were admitted to Mansoura University Hospitals, Egypt, during the pandemic. Patients’ sera were screened for *Toxoplasma* IgG antibodies using ELISA (Roche Diagnostics, Indianapolis, USA), and the gene expression of important immune markers (iNOS, arginase-1, IFN-γ, TNF-α, IL-6, IL-10, and TGF-β) was checked using real-time quantitative PCR. Clinical and laboratory data were obtained from the patients’ medical records.

Results

*Toxoplasma* IgG antibodies were detected in 33 (75%) of patients. None of the studied clinical or laboratory parameters showed any significant changes in relation to toxoplasmosis seroprevalence. Further classification of the patients according to COVID-19 severity and *Toxoplasma* seroprevalence did not reveal any changes related to toxoplasmosis as well.

Conclusion

Our study indicates that latent toxoplasmosis has no effect on the severity of COVID-19.

## Introduction

*Toxoplasma gondii* (*T. gondii*) is an intracellular protozoan that belongs to Apicomplexa. Its prevalence ranges from 20-80% among different countries. The outcome of toxoplasmosis depends on the immune status of the host. Individuals with competent immune systems may exhibit a mild acute disease, but they develop asymptomatic persistent infection, where they continue to harbor the parasites in the nervous system and the muscles, a condition known as latent toxoplasmosis. In this case, the patient is immune to reinfection with low levels of Toxoplasma immunoglobulin (Ig)G [1]; nevertheless, immunodeficiency allows the reactivation of latent stages and renders toxoplasmosis a life-threatening disease [2].

The world has witnessed the coronavirus disease 2019 (COVID-19) pandemic, which resulted from the severe acute respiratory distress syndrome coronavirus 2 (SARS-CoV-2), with global morbidity and mortality. SARS-CoV-2 virus belongs to the family Coronaviridae, which transmits via the respiratory route [3]. After entry, the virus nucleic acids get recognized and activate the innate immune cells [4]. Cytokines in the form of tumor necrosis factor (TNF)-α, interleukin (IL)-18, and type I/III interferons (IFN) are released and activate the adaptive immune response [5]. Mild cases of COVID-19 displayed strong IFN-activated gene expression [6]. Nevertheless, IL-18 intensifies macrophage activation with subsequent decreased release of type I IFN [7]. Besides, auto-antibodies against type I IFN were claimed to cause innate immune dysregulation causing severe disease [8].

Regarding adaptive immunity, B and T cells play crucial roles during SARS-CoV-2 infection, particularly in mild cases [4,9]. Mild/moderate cases exhibited strong functional T helper type 1 (Th1) responses during convalescence [9], and higher levels of CD8 T cells with potent cytotoxic substances [10]. However, severe cases were reported to have high levels of specific T cells of exhausted phenotypes, with lower IFN-γ/ TNF-α ratio [11,12]. Remarkable integrated models connecting the immune response to the disease severity were generated by Sette and Crotty [3], where delayed and poor type I/III IFN-mediated innate immune response was claimed to enhance early proliferation of the virus with the inability to timely-prime the adaptive immune system, leading to severe disease.

Interestingly, similarities exist between the immune response to SARS-CoV-2 and *T. gondii* [13]. Upon IFN-γ stimulation, immunity-related GTPases (IRGs) are recruited to the parasitophorous vacuolar membrane, causing its perforation and destruction of the parasite [14]. CD4 T cells control the acute stage of infection through IFN-γ-mediated activation of macrophages, while CD8 T cells keep infection in the chronic stage under control [15]. The role of CD8 T cells during chronic toxoplasmosis is reliant on the integrity and functionality of CD4 T cells, which display exhaustion, and consequently, CD8 T cells become dysfunctional [16]. To assure survival and replication inside the host, *T. gondii* was reported to secrete a variety of substances that impede the IFN-γ/IRG pathway, with long-term defects in the number and function of naïve T cells [17]. Interestingly, Weeratunga et al. [18] reported that Toxoplasma dense granule protein (GRA)-7 can inhibit the replication of several viruses, both in vivo and in vitro, by enhancing the release of type I IFN.

Due to the complex interplay between the evasion mechanisms generated by parasites and the opposing actions taken by the host immune system, we hypothesized that toxoplasmosis could influence the severity of COVID-19. Toxoplasma antibodies were tested in the sera of 44 hospitalized COVID-19 patients. Gene expression of inducible nitric oxide synthase (iNOS), arginase-1, IFN-γ, TNF-α, IL-10, IL-6, and transforming growth factor (TGF)-β was checked in patients’ sera. Clinical and laboratory parameters were compared between Toxoplasma seropositive and seronegative patients. Identifying such a relationship could improve the outcome of COVID-19 through focusing on early diagnosis and treatment of toxoplasmosis, or the use of Toxoplasma-derived effectors in vaccine development against COVID-19.

## Materials and methods

Type of study

This is a pilot study conducted on patients diagnosed with COVID-19 and admitted to the Isolation Department, Mansoura University Hospitals, Mansoura, Egypt, during the period of March 1st to October 1st, 2021. Approval of the Mansoura Faculty of Medicine Institutional Review Board (MFM-IRB) was obtained (No. R.21.02.1201-2021/02/11) for the study. A consent form was signed by all participants.

Sample size calculation

Based on a previous study by Montazeri et al. [19], we hypothesized that at least 75% of COVID-19 patients have *T. gondii* infection. We also hypothesized that inflammatory markers are significantly higher in those with positive *T. gondii* infection with a large effect size, d = 0.9. Group sample sizes of 11 negative *T. gondii* infection and 33 positive *T. gondii* infection achieve 81.6% power to reject the null hypothesis of zero effect size when the population effect size is 0.90 and the significance level (α) is 0.050 using a one-sided two-sample equal-variance t-test. The sample size was calculated by using G*Power software, version 3.1.9.7 (www.gpower.hhu.de/).

Samples collection

**Figure 1:**
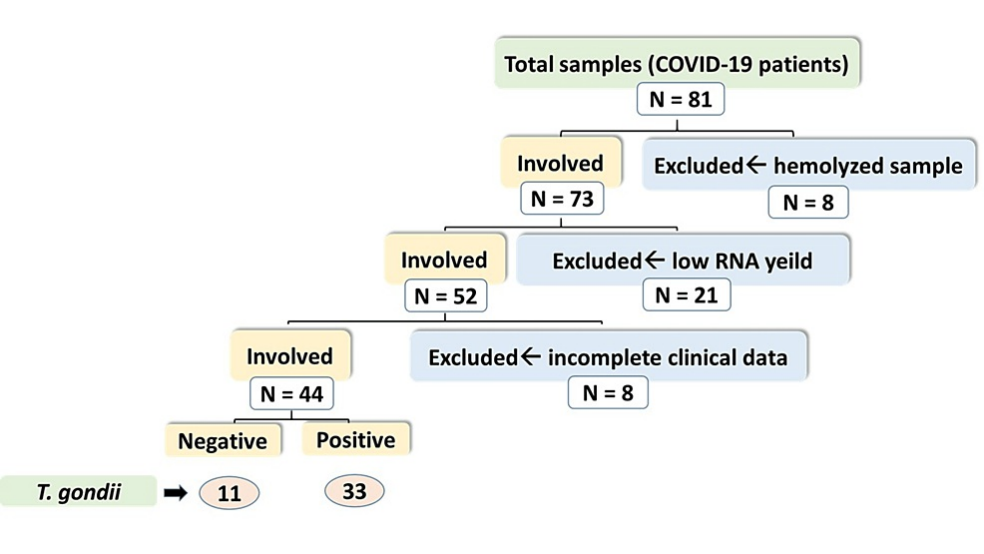
Flow chart of the patient samples enrolled in the study

Eight milliliters of blood were collected from 81 patients, 4 ml in EDTA-containing blood collection tubes and 4 ml were used to prepare serum. After the exclusion of hemolyzed samples, samples with low RNA yield, and samples belonging to patients with incomplete clinical data, 44 samples were subjected to the Toxoplasma antibody test, as elucidated in the flow chart (Figure 1). Gene expression of different cytokines was measured by real-time polymerase chain reaction (PCR). In addition, clinical and laboratory data such as complete blood count (CBC), serum alanine transaminase (ALT), and C-reactive protein (CRP) levels were obtained from the patients’ medical records.

Detection of Toxoplasma IgG antibodies

According to the manufacturer's instructions, the Toxoplasma IgG test was carried out using Elecsys Toxo IgG kits (Roche Diagnostics, Indianapolis, USA). Cobas e 411 analyzer (Roche Diagnostics, Indianapolis, USA) was used.

Detection of cytokines mRNA expression by real-time PCR

Blood samples were treated with RBCs lysis buffer, then centrifuged till separation of the white blood cells. Total RNA extraction was performed utilizing trizol reagent in accordance with the manufacturer′s specifications. The RNA concentration and purity were checked by Thermo Scientific™ NanoDrop™ One (Thermo Fisher Scientific, Waltham, USA). Reverse transcription of 1 ug of RNA was done using SensiFAST™ cDNA Synthesis Kit (Bioline, UK) on Applied Biosystems Proflex Thermal Cycler (Thermo Fisher Scientific, Waltham, USA). cDNA templates were amplified using a real-time PCR instrument (Azure Cielo 6, Azure, USA). The amplification reaction contained 20 μl total volume mixture (10 μl of Bioline SYBR green PCR Master Mix (Bioline, UK), 1 μl of cDNA template, 2 μl (10 pmol/μl) gene primer, and 7 μl of nuclease-free water [20]. β-actin was used as a housekeeping gene. The sequences of the used primer pairs are supplied in Table 1. The primer sets were designated using Primer3Plus software [21], and primer specificity was determined using the Primer-BLAST program [22]. Primer sets were synthesized by Vivantis (Vivantis Technologies, Malaysia).

**Table 1:** The sequence of human primers used in qRT-PCR analysis qRt-PCR: Quantitative reverse transcription polymerase chain reaction

Gene	Sequence	Product size	RefSeq
Inducible nitric oxide synthase (iNOS)	Forward primer: CACCATCCTGGTGGAACTCT Reverse primer: TCCAGGATACCTTGGACCAG	114 bp	NM_000625.4
Arginase 1 (Arg-1)	Forward: GCCAAGTCCAGAACCATAGG Reverse: CAAGCAGACCAGCCTTTCTC	106 bp	NM_000045.4
Interleukin 6 (IL-6)	Forward: GAGGAGACTTGCCTGGTGAA Reverse: GCTCTGGCTTGTTCCTCACT	104 bp	NM_000600.5
Interleukin 10 (IL-10)	Forward: CCAAGCTGAGAACCAAGACC Reverse: GCATTCTTCACCTGCTCCAC	147 bp	NM_000572.3
Tumor necrosis factor-α (TNF-α)	Forward: ATCAGAGGGCCTGTACCTCA Reverse: GATGGCAGAGAGGAGGTTGA	139 bp	NM_000594.4
Transforming growth factor-beta (TGF-β)	Forward: AGCTCCACGGAGAAGAACTG Reverse: GTCCAGGCTCCAAATGTAGG	141 bp	NM_000660.7
Interferon Gamma (IFN-γ)	Forward: TGTCGCCAGCAGCTAAAAC Reverse: GCAGGCAGGACAACCATTAC	90 bp	NM_000619.3
β-actin	Forward: GTGGCCGAGGACTTTGATTG Reverse: GTGGGGTGGCTTTTAGGATG	104 bp	NM_001101.5

Relative gene expression levels were represented as ΔCt = Ct target gene - Ct housekeeping gene; fold change of gene expression was calculated according to the 2−ΔΔCT method [23]. PCR products were run on a 3% agarose gel and visualized on a UV transilluminator (OWI Scientific, France). Then, the gels were photographed using the Azure 600 gel documentation system (Azure, USA).

Statistical analysis

Data were entered and analyzed using IBM SPSS Statistics for Windows, Version 25.0 (IBM Corp., Armonk, NY). Qualitative data were expressed as frequency and percentage and compared by the Chi-Square test. Quantitative data were initially tested for normality using Shapiro-Wilk’s test, with data being normally distributed if p>0.050. Quantitative data were expressed as the median and interquartile range (IQR) as it was not normally distributed. Quantitative data between the two groups were compared by the non-parametric Mann-Whitney U test. Kruskal-Wallis H test with pairwise comparisons was used to compare quantitative data when not normally distributed between the four study groups. Spearman's correlation was used as a measure of the strength and direction of the association/relationship between two continuous variables. For any of the used tests, results were considered statistically significant if p ≤ 0.050. Appropriate charts were used to graphically present the results whenever needed.

## Results

Descriptive data of COVID-19 cases

This study included 44 COVID-19 patients; 11 (25%) patients were found to be Toxoplasma negative, while the other 33 (75%) were Toxoplasma positive. The Toxoplasma negative group involved six males and five females, and the median age was 63 years (interquartile range 50-69 years), while the Toxoplasma positive group involved 25 males and eight females, and the median age was 64 years (interquartile range 50-75.5 years). Complete patients′ characteristics and laboratory parameters in the two study groups were compared with non-significant differences, as listed in Table 2.

**Table 2:** Patients′ characteristics and laboratory parameters in Toxoplasma IgG seronegative versus Toxoplasma IgG seropositive groups TLC: total leukocytic count. NLR: neutrophils to lymphocytes ratio. ALT: alanine transaminase. CRP: C reactive protein. RR: respiratory rate. HR: heart rate. HTN: hypertension. SPO2-RA: peripheral oxygen saturation on room air. SPO2-O2: peripheral oxygen saturation on oxygen device. LOS: length of hospital stay. RBS: random blood sugar. MV: mechanical ventilation. ICU: intensive care unit. DM: diabetes mellitus. DVT: deep vein thrombosis. Data are presented as count (percent) or median (interquartile range). P value by Chi-Square Test (X2) a or Mann-Whitney U Test b. *P* value ≤ 0.05 is considered significant.

Variable	Group		Test of significance	
	Toxoplasma negative (n = 11)	Toxoplasma positive (n = 33)		
Sex			X^2^	p-value^ a^
Male	6 (19.4%)	25 (80.6%)	1.783	^a ^0.265
Female	5 (38.5%)	8 (61.5%)		
Characteristic			Z	p-value ^b^
Age (years)	63 (50-69)	64 (50-75.5)	159.5	0.556
TLC (*10^3^/μl blood)	8.7 (7.9 – 11.1)	9.2 (7.00 – 13.75)	170.5	0.769
Neutrophils (*10^3^/μl blood)	1 (1- 7.1)	1 (1-8.15)	166	0.689
Lymphocytes (*10^3^/μl blood)	1.3 (0.72 – 1.7)	1.2 (0.8-2.0)	165.5	0.669
NLR	0.91 (0.59 – 4.73)	1.25 (0.71 – 5.45)	168.5	0.728
Platelet count (*10^3^/μl blood)	221 (138-292)	220 (161-260)	174.5	0.852
ALT (IU/L)	31 (25-98)	35 (25-53.5)	175.5	0.873
CRP (mg/dL)	92.08 (48 – 96)	83.14 (48-96)	167.000	0.708
RR	22 (20 – 23)	24 (22 – 28)	-1.748	0.083
HR	90 (70 – 99)	87 (78 – 96)	-0.122	0.915
HTN				
Yes	0 (0%)	3 (9.1%)		0.562^ a^
No	11 (100%)	30 (90.9%)		
SPO_2_-RA	86 (80 – 94)	90 (87.5 – 94)	-0.966	0.334
SPO_2_-O_2 _	98 (95 – 99)	97 (95.5 – 99)	-0.137	0.891
O_2 _device				
No	0 (0%)	2 (6.1%)		0.839 ^a^
Simple mask	6 (54.5%)	17 (51.5%)		
Nasal cannula	4 (36.4%)	10 (30.3%)		
Reservoir mask	1 (9.1%)	4 (12.1%)		
LOS	8 (5 – 11)	9 (6.5 – 17)	-1.116	0.264
RBS (mg/dL)	125 (100 – 174)	150 (122 – 220)	-1.275	0.202
MV				
Yes	2 (18.2%)	6 (18.2%)		0.657 ^a^
No	9 (81.8%)	27 (81.8%)		
Admission to ICU				
Yes	5 (45.5%)	15 (45.5%)		0.634 ^a^
No	6 (54.5%)	18 (54.5%)		
Comorbidity				
No	7 (63.6%)	20 (60.6%)		0.500^ a^
DM	0 (0%)	4 (12.2%)		
Renal	0 (0%)	2 (6.1%)		
Hepatic	2 (18.2%)	2 (6.1%)		
Thyroid	1 (9.1%)	1 (3%)		
DVT	1 (9.1%)	0 (0%)		
Hernia	0 (0%)	1 (3%)		
Gout	0 (0%)	1 (3%)		
Cardiac	0 (0%)	1 (3%)		
Oncology	0 (0%)	1 (3%)		

Toxoplasma seroprevalence revealed no difference regarding macrophages, T cell polarization, or Tregs activation

**Figure 2:**
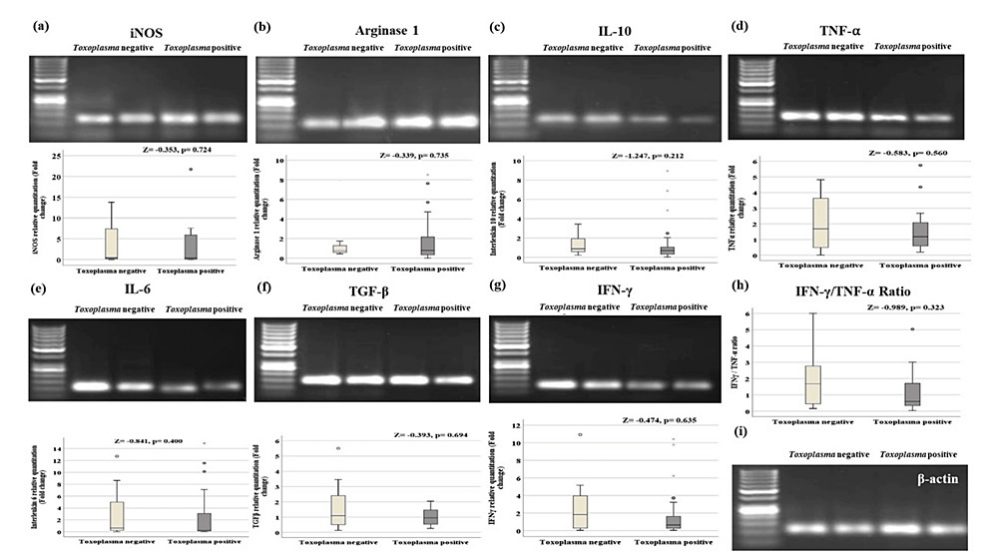
Gel electrophoresis and gene expression of the studied genes in Toxoplasma negative and Toxoplasma positive COVID-19 patients by quantitative real-time PCR All genes expressed non-significant changes in both groups. (a) iNOS qPCR product (114 bp). (b) Arginase 1 qPCR product (106 bp). (c) IL-10 qPCR product (147 bp). (d) TNF-α qPCR product (139 bp). (e) IL-6 qPCR product (104 bp). (f) TGF-β qPCR product (141 bp). (g) IFN-γ qPCR product (90 bp). (h) IFN-γ/ TNF-α ratio. (i) β-actin qPCR product (104 bp). β-actin was used as a housekeeping gene. Data are presented as median (interquartile range) of gene expression fold changes, p-value by Mann-Whitney U test.

Gene expression of iNOS and arginase-1 was measured to explore the switch of macrophage polarization towards M1 or M2. Pro-inflammatory cytokines characteristic of type 1 response, in the form of IL-6, TNF-α, and IFN-γ, were explored, and IL-10 was checked as an anti-inflammatory cytokine characteristic of type 2 response. TGF-β was explored as a marker for T regulatory cell (Tregs) activity. As shown in Figure 2, there are non-significant differences in the studied genes between Toxoplasma negative and Toxoplasma positive groups.

Immune markers displayed stronger correlations in the Toxoplasma positive group compared to the Toxoplasma negative group

The levels of the seven studied genes were tested for correlation with each other in Toxoplasma-negative cases involved in this study (Table 3). iNOS displayed positive correlation with IL-6 (rs = 0.680, p = 0.021) and IFN-γ (rs = 0.789, p = 0.004). IL-10 did not exhibit significant correlations with any of the studied markers. TNF-α showed a positive correlation with IFN-γ (rs = 0.688, p = 0.019) and TGF-β (rs = 0.715, p = 0.013). In addition, IL-6 showed a positive correlation with IFN-γ (rs = 0.765, p = 0.006).

**Table 3:** Correlations of gene expression in Toxoplasma IgG negative cases Ig: Immunoglobulin. iNOS: inducible nitric oxide synthase. IL-10: interleukin-10. TNF-α: tumor necrosis factor-α. IL-6: interleukin-6. TGF-β: transforming growth factor-β. IFN-γ: interferon-γ. RQ = Relative quantitation (Fold change). Rs = Spearman′s correlation coefficient. Bold values = significant p value (≤ 0.050).

Parameter		iNOS RQ	Arginase 1 RQ	IL-10 RQ	TNF-α RQ	IL-6 RQ	TGF-β RQ	IFN-γ RQ
iNOS RQ	r_s_	-	0.267	-0.239	0.189	0.68	0.018	0.789
	P	-	0.428	0.48	0.579	0.021	0.957	0.004
Arginase 1 RQ	r_s_	0.267	-	0.287	0.347	0.11	0.159	0.351
	P	0.428	-	0.392	0.296	0.748	0.64	0.29
IL-10 RQ	r_s_	0.239	0.287	-	0.191	0.096	0.464	-0.109
	P	0.48	0.392	-	0.573	0.78	0.151	0.75
TNF-α RQ	r_s_	0.189	0.347	0.191	-	0.584	0.715	0.688
	P	0.579	0.296	0.573	-	0.059	0.013	0.019
IL-6 RQ	r_s_	0.68	0.11	0.096	0.584	-	0.51	0.765
	P	0.021	0.748	0.78	0.059	-	0.109	0.006
TGF-β RQ	r_s_	0.018	0.159	0.464	0.715	0.51	-	0.318
	P	0.957	0.64	0.151	0.013	0.109	-	0.34
IFN-γ RQ	r_s_	0.789	0.351	-0.109	0.688	0.765	0.318	-
	P	0.004	0.29	0.75	0.019	0.006	0.34	-

Regarding Toxoplasma positive cases, iNOS displayed a negative correlation with IL-10 (rs = -0.420, p = 0.015), a positive correlation with TNF-α (rs = 0.440, p = 0.010), and a strong positive correlation with IL-6 (rs = 0.833, p < 0.001) and IFN-γ (rs = 0.652, p < 0.001). Arginase-1 showed strong positive correlation with IL-10 (rs = 0.563, p = 0.001) and positive correlation with TGF-β (rs = 0.366, p = 0.036). IL-10 displayed negative correlation with IL-6 (rs = -0.435, p = 0.011). TNF-α exhibited a strong positive correlation with IL-6 (rs = 0.679, p < 0.001) and IFN-γ (rs = 0.671, p < 0.001), and a positive correlation with TGF-β (rs = 0.383, p = 0.028). In addition, IL-6 expressed a strong positive correlation with IFN-γ (rs = 0.811, p < 0.001), as shown in Table 4.

**Table 4:** Correlations of gene expression in Toxoplasma IgG positive cases iNOS: inducible nitric oxide synthase. IL-10: interleukin-10. TNF-α: tumor necrosis factor-α. IL-6: interleukin-6. TGF-β: transforming growth factor-β. IFN-γ: interferon-γ. RQ = Relative quantitation (Fold change). Rs = Spearman′s correlation coefficient. Bold values = significant p value (≤ 0.050).

Parameter		iNOS RQ	Arginase 1 RQ	IL-10 RQ	TNF-α RQ	IL-6 RQ	TGF-β RQ	IFN-γ RQ
iNOS RQ	r_s_	-	-0.233	-0.42	0.44	0.833	-0.01	0.652
	P	-	0.192	0.015	0.01	<0.001	0.957	<0.001
Arginase 1 RQ	r_s_	-0.233	-	0.563	-0.199	-0.265	0.366	-0.187
	P	0.192	-	0.001	0.267	0.135	0.036	0.297
IL-10 RQ	r_s_	-0.42	0.563	-	-0.314	-0.435	0.09	-0.25
	P	0.015	0.001	-	0.075	0.011	0.617	0.16
TNF-α RQ	r_s_	0.44	-0.199	-0.314	-	0.679	0.383	0.671
	P	0.01	0.267	0.075	-	<0.001	0.028	<0.001
IL-6 RQ	r_s_	0.833	-0.265	-0.435	0.679	-	0.105	0.811
	P	<0.001	0.135	0.011	<0.001	-	0.561	<0.001
TGF-β RQ	r_s_	-0.01	0.366	0.09	0.383	0.105	-	0.219
	P	0.957	0.036	0.617	0.028	0.561	-	0.221
IFN-γ RQ	r_s_	0.652	-0.187	-0.25	0.671	0.811	0.219	-
	P	<0.001	0.297	0.16	<0.001	<0.001	0.221	-

Moderate and severe COVID-19 displayed non-significant variations between Toxoplasma negative and positive patient groups

Patients were further classified according to the severity of COVID-19, based on the criteria approved by the China National Health Commission [24], which considers patients having severe COVID-19 disease if the respiratory rate (RR) ≥ 30/ min or saturation of oxygen in the peripheral blood (SpO2) ≤ 93%. On applying these criteria, and among the 11 Toxoplasma negative patients, we identified three (27%) cases with moderate disease and eight (73%) patients with severe disease. Similarly, among the 33 Toxoplasma positive patients, nine (27%) of them were distinguished as having moderate disease, and 24 (73%) of them with severe disease. The number of patients with associated hypertension was significantly higher in the Toxoplasma positive moderate group (p = 0.02). SpO2 on room air (RA) and the requirement of ICU admission later during hospitalization were statistically higher in the severe groups than in the moderate groups regardless of Toxoplasma seroprevalence (p < 0.001, p = 0.001, respectively), as described in Table 5.

**Table 5:** Clinical and laboratory data from a perspective of COVID-19 severity and Toxoplasma seroprevalence TLC: total leukocytic count. NLR: neutrophils to lymphocytes ratio. ALT: alanine transaminase. CRP: C reactive protein. RR: respiratory rate. HR: heart rate. HTN: hypertension. SPO2-RA: peripheral oxygen saturation on room air. SPO2-O2: peripheral oxygen saturation on oxygen device. LOS: length of hospital stay. RBS: random blood sugar. MV: mechanical ventilation. ICU: intensive care unit. DM: diabetes mellitus. DVT: deep vein thrombosis. Data are presented as count (percent) or median (interquartile range). P value by Chi-Square Test (X2) a or Kruskal-Wallis H Test b. *P* value ≤ 0.05 is considered significant. Interquartile range could not be computed with some variables in the first group.

Variable	Group				Test of significance	
	Toxoplasma negative/ moderate COVID-19 (n=3)	Toxoplasma negative/ severe COVID-19 (n=8)	Toxoplasma positive/ moderate COVID-19 (n=9)	Toxoplasma positive/ severe COVID-19 (n=24)		
Sex					X^2^	p-value^ a^
Male	1 (33.3%)	5 (62.5%)	7 (77.8%)	18 (75%)	-	0.425^ a^
Female	2 (66.7%)	3 (37.5%)	2 (22.2%)	6 (25%)		
Characteristic					H	p-value^ b^
Age (years)	50 (26-58)	67 (59.25 – 69)	65 (46.5 – 75)	61.5 (49.5 – 72.75)	3.577	0.311
TLC (*10^3^/μl blood)	8.3 (7.5 – 8.7)	9.1 (8.1 – 14.85)	13.5 (7.7-14.9)	8.55 (5.58 – 11.91)	3.210	0.360
Neutrophils (*10^3^/μl blood)	1 (1 – 5.6)	1 (1 – 7.47)	4 (1 – 11.5)	1 (1 – 7.25)	1.505	0.681
Lymphocytes (*10^3^/μl blood)	1.3 (0.5 – 1.7)	1.4 (0.82 – 1.78)	1.8 (1.26-2.13)	1.15 (0.8 – 1.83)	5.229	0.156
NLR	2	0.84 (0.56–9.09)	1.67 (0.57–5.4)	1.13 (0.83 – 5.97)	0.157	0.984
Platelet count (*10^3^/μl blood)	160 (8 - 292)	250.5 (153.25-308)	227 (120.5-359.5)	219.5 (166-241.5)	1.152	0.765
ALT (IU/L)	37	28 (22.75 – 82.5)	35 (19.5 – 50)	34 (25 – 53.75)	1.351	0.717
CRP (mg/dL)	92.08	71.04 (41.73 – 95.02)	83.14 (48- 120.5)	75.07 (30 – 96)	1.242	0.743
RR	22	22 (20 – 22.75)	22 (20 – 25.5)	25.5 (22.5 – 29.5)	7.406	0.060
HR	70	91 (84 – 100.5)	92 (80 – 100.5)	86.5 (73.75 – 90)	6.144	0.105
HTN						
Yes	0 (0%)	0 (0%)	3 (33.3%)	0 (0%)		0.026^ a^
No	3 (100%)	8 (100%)	6 (66.7%)	24 (100%)		
SPO_2_-RA	96	86 (72.5 –98.75)	94 (94 – 96)	89.5 (86 – 91)	27.482	<0.001^ b^
SPO_2_-O_2_	100	96 (92.75-98)	98 (96.5-99)	97 (95-99)	7.573	0.056^ b^
O_2 _device						
No	0 (0%)	0 (0%)	2 (22.2%)	0 (0%)	-	0.671^ a^
Simple mask	2 (66.7%)	4 (50%)	4 (44.54%)	13 (54.2%)		
Nasal cannula	1 (33.3%)	3 (37.5)	2 (22.2%)	8 (33.3%)		
Reservoir mask	0 (0%)	1 (12.5%)	1 (11.1%)	3 (12.5%)		
LOS	7	8 (5 – 12.5)	7 (4.5 – 17)	9 (7 – 17.75)	2.842	0.417^ b^
RBS	112	154 (106 – 177)	210 (122- 230)	146.5 (115.75-199)	3.931	0.269^ b^
MV						
Yes	0 (0%)	2 (25%)	0 (0%)	6 (25%)	-	0.375^ a^
No	3 (100%)	6 (75%)	9 (100%)	18 (75%)		
Admission to ICU						
Yes	0 (0%)	5 (62.5)	0 (0%)	15 (62.5%)	-	0.001^ a^
No	3 (100%)	3 (37.5%)	9 (100%)	9 (37.5%)		
Comorbidity						
No	2 (66.7%)	5 (62.5%)	5 (55.6%)	15 (62.5%)	-	0.563^ a^
DM	0 (0%)	0 (0%)	1 (11.1%)	3 (12.5%)		
Renal	0 (0%)	0 (0%)	2 (22.2%)	0 (0%)		
Hepatic	1 (33.3%)	1 (12.5%)	0 (0%)	2 (8.3%)		
Thyroid	0 (0%)	1 (12.5%)	0 (0%)	1 (4.2%)		
DVT	0 (0%)	1 (12.5%)	0 (0%)	0 (0%)		
Hernia	0 (0%)	0 (0%)	1 (12.5%)	0 (0%)		
Gout	0 (0%)	0 (0%)	0 (0%)	1 (4.2%)		
Cardiac	0 (0%)	0 (0%)	0 (0%)	1 (4.2%)		
Oncology	0 (0%)	0 (0%)	0 (0%)	1 (4.2%)		

Toxoplasma seropositivity displayed non-significant changes in the studied cytokines in different COVID-19 severity groups

**Figure 3:**
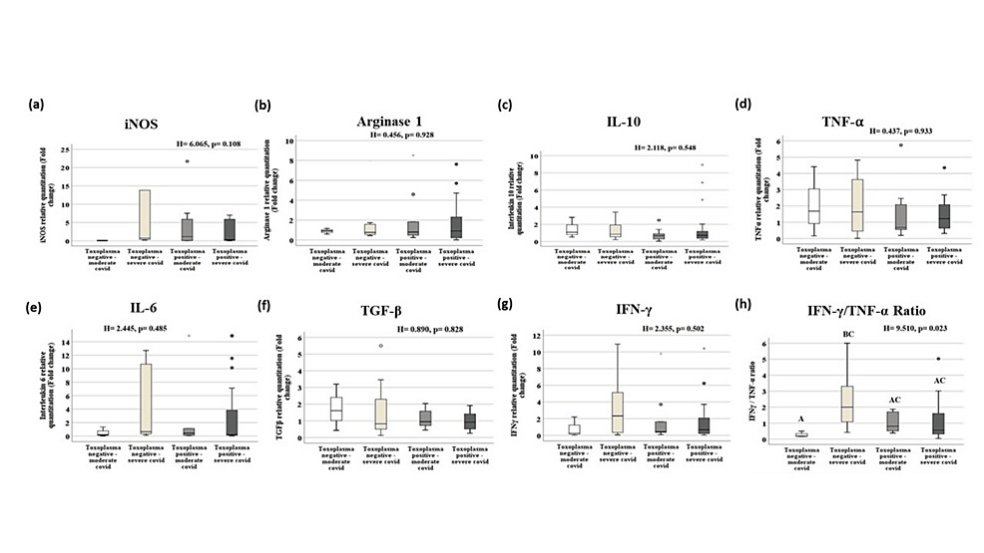
Gene expression of the studied genes in Toxoplasma negative and Toxoplasma positive moderate and severe groups by quantitative real-time PCR. All immune markers expressed non-significant changes in the four studied groups. Only the IFN- γ/TNF-α ratio displayed a significant increase in the Toxoplasma negative severe group compared to the Toxoplasma negative moderate group (p = 0.02) (h). Data are presented as median (interquartile range) of gene expression fold changes, p-value by Kruskal-Wallis H test. Pairwise comparison when p is significant is presented as capital letters; similar letters = statistically insignificant difference, different letters = statistically significant difference.

As shown in Figure 3, the classification of the patients according to COVID-19 severity and Toxoplasma seroprevalence did not reveal any significant differences in the studied cytokines. Notably, IFN-γ/ TNF-α ratio was significantly lower in the Toxoplasma negative moderate group compared to the Toxoplasma negative severe group (P = 0.023).

## Discussion

The interplay between parasitic infections and other diseases has been an issue of discussion in many studies, owing to the immune modulation caused by parasites, for purposes of immune evasion and subsequent establishment of chronic infections. In this study, we tried to unravel the relationship between COVID-19 disease and latent toxoplasmosis. We collected clinical, laboratory data, and blood samples from COVID-19 patients admitted to the Isolation Department, Mansoura University Hospitals, Mansoura University, Egypt. The sera were checked for the Toxoplasma IgG antibodies and different immune markers. *T. gondii* tested positive in 75% of cases. It was not possible to include COVID-19 negative controls or patients with mild COVID-19 in the study because, at the time of sample collection quarantine at homes was applied, rendering access to healthy controls and mild COVID-19 patients difficult. Also, vaccination against SARS-CoV-2 would make it difficult to distinguish between previously infected and vaccinated personnel through antibody tests.

The prevalence of toxoplasmosis in the studied patients was 75%, which looks higher than in general populations. Studies have reported the prevalence of toxoplasmosis among the general Egyptian population up to 42.5%, and up to 67.4% among healthy blood donors, with higher prevalence in Northern governorates [25]. In Dakahlia, one of the Northern governorates, where we conducted our study, a couple of studies reported Toxoplasma prevalence to be 59.6% and 67.4% [26,27]. Given that the mean age in our study is above 60 and Toxoplasma prevalence increases in the elderly [28], we suggest that the prevalence of toxoplasmosis in our study is not different from healthy controls with matched age. It is worth mentioning that increased age and associated comorbidities are linked to augmented severity of COVID-19. Aging is associated with less number of naïve T cells, with reduced proliferative potential, thus coordinated T cell response is not achieved with delayed humoral response, resulting in higher viral load. Innate response tries to compensate for the delayed adaptive response. Hyper-activation of the innate system causes cytokine storm, lung immunopathology, and end-stage disease [3].

Based on the blood count-related severity parameters such as total leukocytic count, neutrophil, lymphocyte, and platelet counts, as well as neutrophil to lymphocyte ratio (NLR), CRP, and ALT, Toxoplasma seropositivity was not associated with any significant change in the listed factors. Further, none of the studied genes exhibited significant change between Toxoplasma seropositive and seronegative groups. Remarkably, significant correlations were evident in the Toxoplasma positive group, which did not appear in the Toxoplasma negative group, like that between arginase-1 and IL-10, and that between iNOS and TNF-α. Also, similar correlations displayed in both groups showed higher significance in the Toxoplasma positive group, like that between iNOS and both IL-6 and IFN-γ. For this, we speculate that macrophage polarization is more dominant in Toxoplasma positive patients, while in Toxoplasma negative patients, other cells together with macrophages contribute significantly to cytokines production. This may be attributed to the “trained immunity” observed in latent toxoplasmosis, which is a form of innate immune memory, like that noticed in monocytes derived from Toxoplasma seropositive patients, which were phenotypically different from those derived from seronegative controls, leading to enhanced response to ex vivo Toxoplasma infection [29]. In the referred study, the authors suggested that this innate memory may not be exclusive to Toxoplasma, and may include heterologous pathogens. Here, even if the trained immunity had a role, it was not sufficient to improve or worsen the outcome of COVID-19 in Toxoplasma seropositive patients.

To further confirm our findings, we classified the patients according to COVID-19 severity and Toxoplasma seroprevalence. Intriguingly, severe COVID-19 was distinguished in 73% of each of the Toxoplasma negative and positive groups. The requirement of ICU admission later in the course of COVID-19 disease was statistically higher in the severe than in the moderate groups regardless of Toxoplasma seroprevalence (p = 0.001) (Table 5). Also, SpO2-RA was significantly different among the moderate and the severe groups irrespective of Toxoplasma seroprevalence, and this is predictable because SpO2 and ICU admission were used as key criteria to signify the severity of COVID-19. All other clinical and laboratory parameters displayed non-significant changes between Toxoplasma negative and positive patients in either the moderate or the severe groups. Similar to the previous classification, none of the studied genes exhibited significant differences among Toxoplasma positive and negative moderate or severe groups, confirming that latent toxoplasmosis does not affect the severity of COVID-19. Unexpectedly, the IFN-γ/ TNF-α ratio was significantly lower in the Toxoplasma negative moderate group compared to the Toxoplasma negative severe group, indicating a worse prognosis in the former group [4], which may have resulted from the small number of patients in the former group.

Several studies were conducted in different countries to explore the link between latent toxoplasmosis and COVID-19 with debatable results. In line with our findings, two studies were conducted in Iran. They reported no link between latent toxoplasmosis and COVID-19 severity, although a high rate of toxoplasmosis was noticed in different COVID-19 severity categories [19,30]. A third study was also done in Iran and concluded that neither acute nor latent toxoplasmosis are risk factors for COVID-19 [31]. Similarities between the immune response against both SARS-CoV-2 and Toxoplasma are suggested to abolish the influence of toxoplasmosis on COVID-19 outcomes. Also, Toxoplasma secretes a cocktail of effectors; each effector possesses either immune-stimulatory or inhibitory criteria, as reviewed by Tomita et al. [32]. In the case of superimposed SARS-CoV-2 infection, the immune-stimulatory effects of GRA7, GRA15II, GRA24, and GRA25 may have neutralized the immune-inhibitory effects of the other effectors; therefore, the impact of toxoplasmosis on COVID-19 could not be recognized.

An internet-based study was done on the Czech and Slovac populations and concluded that latent toxoplasmosis is a risk factor for COVID-19 acquisition and severity [33]. The difference between the findings of this study and ours may be due to different study types. Participants were recruited via social networks; besides, toxoplasmosis was self-informed, and patients with severe COVID-19 did not participate in the referred study. Another study was performed in Egypt and concluded that latent toxoplasmosis causes increased COVID-19 severity. They attributed this link to increased expression of programmed death (PD)-1 receptor on T lymphocytes from Toxoplasma positive patients [34]. We speculate that different times of sample collection during the pandemic between the latter study and ours, with different COVID-19 variants and *T. gondii* genotypes may have led to different findings. Although both *T. gondii* and SARS-CoV-2 upregulate PD-1 on antigen-specific T cells, it is not confirmed yet whether programmed death ligand (PDL)-1 expressed on Toxoplasma-infected cells could cross-react with PD-1 receptors on naïve or SARS-CoV-2 antigen-specific T cells [35].

In contrast to the above-mentioned studies, a negative covariation between toxoplasmosis and COVID-19 was established by Jankowiak et al. [36]. This study linked the prevalence of toxoplasmosis, as an indicator of hygiene, in pregnant females in over 86 countries, to the date of the first reported COVID-19 case in each country, as an indicator of COVID-19 susceptibility. Nevertheless, the authors remarked that toxoplasmosis may serve as a false factor in this covariation and the link is mostly mediated by the gross domestic product (GDP) per capita and time factors. Another study was conducted in Egypt [37] and reported higher rates of parasitic infections in mild cases of COVID-19 versus severe cases, with *T. gondii* representing the most prevalent infection. High levels of IFN-γ were detected in parasite-infected compared to parasite-free patients, in moderate and severe COVID-19. However, this study combined the effects of several parasitic infections together and was not focused on *T. gondii*.

We acknowledge that patients were admitted to the hospital at different stages of the disease, which made it difficult to have homogenous patient groups. We did not check Toxoplasma IgM to exclude possible acute toxoplasmosis. We recommend conducting a similar study, whenever possible, on a larger scale. Also, the establishment of murine and in vitro models of co-infection will be valuable.

## Conclusions

Latent toxoplasmosis is common among moderate/ severe COVID-19 patients. Toxoplasma IgG seropositivity did not reveal any significant effect on the clinical consequences or the laboratory criteria of COVID-19 patients. Also, immune markers of T cell/macrophage polarization and Tregs activation did not exhibit significant differences between Toxoplasma positive versus Toxoplasma negative patient groups. More and higher significances were observed only in the correlations among immune markers within the Toxoplasma positive group when compared to the Toxoplasma negative group, suggesting a role of trained immunity; however, these variations were not reflected in individual gene expression.

Further classification of patients with respect to COVID-19 severity and Toxoplasma seroprevalence did not display significant variations in the expression of the same genes or in the clinical outcome. Thus, our study suggests that latent toxoplasmosis has no effect on the severity of COVID-19.
